# Multifaceted amelioration of cutaneous photoageing by (0.3%) retinol

**DOI:** 10.1111/ics.12799

**Published:** 2022-09-06

**Authors:** Kieran T. Mellody, Eleanor J. Bradley, Bezaleel Mambwe, Lindsay F. Cotterell, Orsolya Kiss, Poonam Halai, Zeena Loftus, Mike Bell, Tamara W. Griffiths, Christopher E. M. Griffiths, Rachel E. B. Watson

**Affiliations:** ^1^ Centre for Dermatology Research The University of Manchester & Salford Royal NHS Foundation Trust, Manchester Academic Health Science Centre Manchester UK; ^2^ Manchester Institute for Collaborative Research on Ageing University of Manchester Manchester UK; ^3^ No7 Beauty Company Walgreens Boots Alliance Nottingham UK; ^4^ NIHR Manchester Biomedical Research Centre Manchester University NHS Foundation Trust, Manchester Academic Health Science Centre Manchester UK

**Keywords:** formulation, photodamage, skin barrier, skin physiology/structure

## Abstract

**Background:**

Although retinol skin care products improve the appearance of photoaged skin, there is a need for an effective retinol concentration that provides skin benefits without irritation.

**Objective:**

To compare the efficacy of topical 0.1%, 0.3% and 1% retinol in remodelling the cutaneous architecture in an *in vivo* experimental patch test study, and to determine tolerance of the most effective formulations when used in a daily in‐use escalation study.

**Methods:**

For the patch test study, retinol products were applied under occlusion, to the extensor forearm of photoaged volunteers (*n* = 5; age range 66–84 years), and 3 mm skin biopsies obtained after 12 days. Effects of different retinol concentrations, and a vehicle control, on key epidermal and dermal biomarkers of cellular proliferation and dermal remodelling were compared to untreated baseline. Separately, participants (*n* = 218) recorded their tolerance to 0.3% or 1% retinol over a six‐week, approved regimen, which gradually increased the facial applications to once nightly.

**Results:**

Retinol treatment induced a stepwise increase in epidermal thickness and induced the expression of *stratum corneum* proteins, filaggrin and KPRP. 0.3% retinol and 1% retinol were comparably effective at inducing keratinocyte proliferation in the epidermis, whilst reducing e‐cadherin expression. Fibrillin‐rich microfibril deposition was increased following treatment with 0.3% and 1% retinol (*p* < 0.01); other dermal components remained unaltered (e.g., fibronectin, collagen fibrils, elastin), and no evidence of local inflammation was detected. The in‐use study found that 0.3% retinol was better tolerated than 1% retinol, with fewer and milder adverse events reported (χ^2^(1) = 23.97; *p* < 0.001).

**Conclusions:**

This study suggests that 1% and 0.3% retinol concentrations were similarly effective at remodelling photodamaged skin in an *in vivo* model of long‐term use. Use of 0.3% retinol in the escalation study was associated with fewer adverse reactions when applied daily. Hence, 0.3% retinol may be better tolerated than 1% retinol, thereby allowing longer‐term topical application.

## Graphical abstract

This investigation focusses on changes to the cutaneous anatomy in response to increasing concentrations of retinol (0.1%, 0.3%, and 1% retinol), as compared with baseline and vehicle control‐treated skin. These representative images show retinol‐induced alterations at the skins' barrier and in epidermal architecture. Immunostaining of KPRP at the stratum corneum intensified, the epidermis thickened with quantifiable changes to the intensity of immunostaining for filaggrin. The number of proliferating keratinocytes also increased, as seen using Ki67, a robust marker of proliferation.

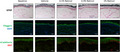

## Résumé

**Contexte:**

Même si les produits de soins pour la peau à base de rétinol améliorent l'apparence de la peau photovieillie, il est nécessaire d'obtenir une concentration efficace de rétinol procurant des bénéfices cutanés sans irritation.

**Objectif:**

Comparer l'efficacité du rétinol à 0.1%, 0.3% et 1% en application locale dans le remodelage de l'architecture cutanée dans une étude d'irritation cutanée in vivo expérimental, et déterminer la tolérance des formulations les plus efficaces lorsqu'elles sont utilisées dans une étude à doses progressives quotidiennes en cours d'utilisation.

**Méthodes:**

Pour l'étude d'irritation cutanée, des produits à base de rétinol ont été appliqués sous occlusion, sur le muscle extenseur de l'avant‐bras de volontaires présentant des signes de photovieillissement (*n* = 5; tranche d'âge: 66 à 84 ans), et des biopsies cutanées de 3 mm ont été obtenues après 12 jours. Les effets des différentes concentrations de rétinol, et d'un véhicule témoin sur les principaux biomarqueurs épidermiques et dermiques de la prolifération cellulaire et du remodelage dermique ont été comparés à ceux observés à une région non traitée. Séparément, les participants (*n* = 218) ont enregistré leur tolérance au rétinol à 0.3% ou 1% au cours d'un schéma posologique approuvé de six semaines, qui a progressivement augmenté les applications faciales à une fois par nuit.

**Résultats:**

Le traitement par rétinol a induit une augmentation progressive de l'épaisseur épidermique, et a induit l'expression des protéines de la couche cornée, la filaggrine et le KPRP. Le rétinol à 0.3% et le rétinol à 1% étaient aussi efficaces pour induire la prolifération des kératinocytes dans l'épiderme, tout en réduisant l'expression de la cadhérine E. Le dépôt de microfibrilles riches en fibrilline a augmenté après un traitement par rétinol à 0.3% et 1% (*p* < 0.001).

**Conclusions:**

Cette étude suggère que les concentrations de rétinol de 1% et 0.3% étaient aussi efficaces pour remodeler la peau photolésée dans un modèle in vivo lors d'une utilisation à long terme. L'utilisation de rétinol à 0.3% dans l'étude à doses progressives a été associée à moins d'effets indésirables lorsqu'il est appliqué quotidiennement. Par conséquent, le rétinol à 0.3% peut être mieux toléré que le rétinol à 1%, permettant ainsi une application topique à plus long terme.

## INTRODUCTION

Chronic exposure to solar ultraviolet radiation results in clinical features associated with cutaneous ageing typically characterized as hypertrophic photoageing, i.e., the presence of wrinkles, coarseness, skin laxity and hyperpigmentation [1]. This hypertrophic photoaged phenotype is underscored by a remodelled cutaneous microenvironment, epidermal thinning and loss of rete ridges [2, 3, 4], dermal solar elastosis [5], loss of fibrillin‐rich microfibrils (FRM) from the papillary dermis [6], and changes to the abundance and cross‐linking of collagen fibrils [1, 7, 8], thereby altering the appearance of the skin and its biophysical properties [9].

Many skincare treatments, from non‐invasive topical applications to minimally invasive micro needling and skin peels, aim to reduce the visible facial signs of skin ageing [10]. All‐*trans* retinoic acid (ATRA) is the gold‐standard treatment prescribed by dermatologists for treating photodamaged skin [11]. Retinol, converted by cells into ATRA, is used commonly in over‐the‐counter topical anti‐ageing cosmetics. It signals to cells via the ‘stimulated by retinoic acid 6’ (STRA6) receptor [12, 13] and the cytoplasmic retinoid‐binding proteins (CRBP) I and II receptors, the latter being the predominant isoform present in skin [14, 15]. Intracellularly, ATRA binds the nuclear retinoic acid receptor (RAR) family that regulate the expression of multiple genes [16, 17, 18, 19, 20].

Mechanisms resulting in photodamage [21] alter epidermal architecture and affect the structural composition of the dermal extracellular matrix (ECM) [22]. Improving the clinical features of hypertrophic photoageing with cosmetics or ‘cosmeceuticals’ requires the inclusion of active compounds, such as retinoids, capable of remodelling cutaneous dermal structure [23]. Retinoids reduce the clinical appearance of wrinkles by thickening the epidermis, stimulating collagen synthesis and restoring the FRM network at the dermal‐epidermal junction (DEJ) [24, 25, 26]. However, retinoids—ATRA, its derivatives and precursors, including retinol—can cause skin irritation, leading to poor patient and/or consumer tolerance [27]. The amount of retinol approved for inclusion in topical cosmetics applications spans a wide range of concentrations (0.05%–1%), guided in each country by local consumer safety recommendations [28]. Agreement on an effective retinol concentration that achieves cosmetic benefits whilst reducing undesirable side effects is lacking.

Here, we conducted the Manchester Patch Test (MPT) assay [25], an *in vivo* protocol that mimics the effects of longer‐term topical application, to compare the effectiveness of a range of retinol concentrations used in cosmetic products (0.1%, 0.3% and 1%) to induce changes in proteins associated with ageing and/or photoageing. These included epidermal biomarkers associated with proliferation (*Ki*67) and barrier integrity (filaggrin, keratin proline‐rich protein [KPRP]) plus deposition of key dermal ECM proteins, known to be altered in photodamaged skin (fibrillar collagens; elastin and FRMs). To determine if the application of 0.3% compared with 1% retinol reduced cutaneous irritation, a six‐week, self‐reporting dermatological study was performed, where the number of retinol formulation applications gradually increased from twice weekly to once nightly, with participants self‐reporting their tolerance profiles.

## MATERIALS AND METHODS

### Tissue sample acquisition and preparation

Five healthy, white (Fitzpatrick skin phototype I‐III) but photoaged volunteers (male = 1: female = 4; age range 66–84 years) were recruited to the study. The formulations used in the patch test study were simple oil‐in‐water emulsions (a gel cream format) comprising water, glycerin, butylene glycol, dimethicone and a preservative system. Retinol formulations (30 μl of the oil‐in‐water emulsion plus retinol at 0.1%, 0.3% and 1% w/w) and its vehicle control were applied to the extensor aspect of photodamaged forearm under Finn chamber occlusion [25]. A further site was occluded without formulation to assess baseline levels of skin biomarkers of damage. Formulations were re‐applied on days 4 and 8, with 3 mm punch biopsies taken from each site on day 12 following 1% lignocaine anaesthesia (*n* = 5 biopsies per volunteer). Biopsies were snap frozen in liquid nitrogen and embedded in optimal cutting temperature compound (Miles Laboratories, Elkhart, IN, USA). All biopsy samples were cryosectioned at 10 μm thickness. The study was conducted in accordance with the principles of the Declaration of Helsinki, with written informed consent (Manchester University Research Ethics Committee reference: 15528).

### Biomarker detection and imaging

Immunohistochemistry was used for detection of a panel of biomarkers associated with skin ageing or photoageing to observe alterations in tissue expression within the epidermis and dermis following topical treatment (for the list of antibodies used, see Table 1). Histological staining for melanin was performed using the modified Warthin‐Starry procedure [29], and epidermal thickness analyzed on hematoxylin and eosin (H&E) stained sections. Picrosirius Red staining, viewed under cross‐polarized light, was used to measure collagen birefringence against the total tissue area. Weigert's resorcin fuchsin was used to detect elastin fibres within the tissue. Immunohistochemistry was performed as previously described [30]. Sections were fixed with 4% paraformaldehyde and hydrated in tris‐buffered saline (TBS; 100 mM Tris, 150 mM NaCl). Sections were permeabilized with 0.5% Triton‐X100 for 10 min prior to antibody incubations for 1 h at room temperature or overnight at 4°C. Sections were washed in TBS, prior to incubation with Alexa Fluor® conjugated, anti‐rabbit or anti‐mouse secondary antibody (Life Technologies, UK). Negative controls were concurrently incubated with a serum‐only block. An Olympus pE‐300 microscope was used to image tissue sections.

**TABLE 1:** Antibodies and suppliers used for the immunohistochemical detection of biomarkers

Target	Supplier	Clone (dilution)
Epidermal		
Filaggrin	Atlas Antibodies	Polyclonal (1:1000)
Keratinocyte proline‐rich protein (KPRP)	Abcam	Polyclonal (1:100)
e‐Cadherin	Abcam	HECD‐1 (1:500)
*Ki*67	Abcam	SP6 (1:500)
Cleaved caspase‐3	Cell Signalling	D3E9 (1:100)
Papillary dermis		
Fibrillin‐rich microfibrils (FRM)	Abcam	11C1.3 (1:250)
Collagen VII	Sigma Aldrich	LH7.2 (1:100)
Pro‐collagen I	Millipore	M‐58 (1:250)
Fibronectin	Leica Biosystems	568 (1:500)
Inflammatory markers		
Macrophages, CD68	Abcam	KP1 (1:60)
M2 macrophages, CD206	Abcam	Polyclonal (1:600)

### Image analysis

Biomarker analysis was performed on stained cryosections using Fiji software [31], and samples were only unblinded by researchers upon completion of analysis. Filaggrin deposition was performed by measuring the mean width of positive staining within the *stratum granulosum*, spanning the length of the epidermis in each field of view. At least six measurements of epidermal depth were performed on each H&E stained section, from the bottom of the *stratum corneum* to the base of the epidermis, excluding the descending protrusions that form the rete ridges. Convolution was determined by dividing the length of the epidermis measured in a straight line by the undulating epidermal length [32]. Global collagen, elastin fibres and dermal fibronectin staining were expressed as percentage positive staining within the tissue area. Melanin was expressed as a ratio of positive staining normalized against the epidermal area. Keratinocyte proliferation was determined by enumerating the number of *Ki*67‐positive epidermal cells in each field of view. Cell counting was also used to determine the number of epidermal cells expressing cleaved caspase‐3; CD68^+^‐ and CD68^+^/CD206^+^‐positive dermal macrophages were also enumerated. Scoring of FRM and procollagen I immunostaining was performed independently by two researchers (KTM, PH) using a 5‐point ordinal scale, as previously described [24]. The average mean score for each site/test area from both independent analyses was reported. Plot profiles were used to measure collagen VII, expressed as area under the curve (AUC).

### Statistical analysis

Differences between the test formulations compared to untreated occluded baseline were analyzed using a repeated‐measures analysis of variance (RM one‐way ANOVA; significance taken as *p* < 0.05). Mean values (± standard error of the mean; SEM) are displayed graphically.

### Consumer tolerance study

Healthy women aged between 35 and 70 years and with self‐perceived photoaged skin, including facial wrinkles and uneven skin tone/pigmentation, were recruited for a six‐week home‐use consumer tolerance study (*n* = 218). The formulations used were again oil‐in‐water emulsions (a gel cream format), comprising those in the patch test, along with bisabolol, peptides and thickeners to improve the formulation aesthetics for an at‐home consumer study. The study was performed in the UK between July 2019 and March 2020. Other eligibility criteria included not having used retinol‐containing products for at least three months prior to the study and be regular users of sun protection factor (SPF)‐containing day creams and night creams.

Cohort one (*n* = 115) applied a formulation containing 0.3% w/w retinol, whilst cohort two (*n* = 103) applied a formulation containing 1% w/w retinol. Participants were required to apply the retinol products at home to the full face, avoiding the delicate eye and lip areas, in the evenings only. Participants were asked to apply two full pumps of the product (supplied in an airless pump pack) at each application equivalent to approximately 0.3 ml. This was followed by application of their usual night‐time moisturizer, which was applied every evening of the study even when retinol was not applied. Their usual SPF day cream was also applied every morning of the study to reduce the risk of photosensitivity, in line with recommended usage instructions for high strength retinol formulations and to help ensure participant safety during the study.

The retinol formulation application regimen and the guidelines for classifying mild, moderate or severe skin reactions were designed (MB TWG ZL), as follows (Tables 2 and 3).

**TABLE 2:** Application regimen in 6‐week tolerance study

Week	Application frequency	Application number
1	Twice a week on non‐consecutive evenings	Application 1+2
2	Twice a week on non‐consecutive evenings	Application 3+4
3	Three times a week on non‐consecutive evenings	Application 5–7
4	Three times a week on non‐consecutive evenings	Application 8–10
5	Every evening	Application 11–17
6	Every evening	Application 18–24

**TABLE 3:** Guidelines for classification of cutaneous skin reactions

Skin reaction classification (adapted from REF)	
1—MILD	Tingling, stinging, tightness, blemishes/spots, peeling, dryness without soreness, slight redness, slight feeling of heat/burning
2—MODERATE	Red, angry and sore to the touch, blind pimples beneath the surface of the skin, large area of dryness, rash
3—SEVERE	Red, angry and sore without touching, eczema‐like and persistent. Reactions can include broken skin, blistering or an extended rash

### Tolerance profile study analysis

Self‐reported tolerance data were gathered from the volunteers on a weekly basis and grouped according to the most severe reaction reported during the course of the study. A participant, for example, reporting three mild reactions and one moderate reaction would be classified as having had a moderate reaction. Participants not reporting reactions were deemed fully tolerant to the retinol formulation. The tolerance profiles of the two cohorts (0.3% and 1% retinol) were compared using a chi‐square statistical test using JMP software.

## RESULTS

Occlusion with retinoids can sometimes induce signs of cutaneous irritation in the form of local erythema, scaling or mild blistering. In this MPT assay, two volunteers showed no signs of cutaneous irritation on retinol treatment, one volunteer had mild erythema in response to 1% retinol only, one volunteer had mild erythema in response to both 0.1% and 0.3% retinol and moderate erythema in response to 1% retinol, and one volunteer had mild blistering in response to 0.3% retinol only. As macrophages are involved in dermal ECM remodelling and skin sensitization, we investigated their infiltration within the dermis in response to retinol occlusion. However, we found no significant change in CD68^+^ cells, a pan‐marker of macrophages or in the number of CD68^+^/CD206^+^ cells, markers of M2 macrophages, compared with the baseline (data not shown).

**FIGURE 1:**
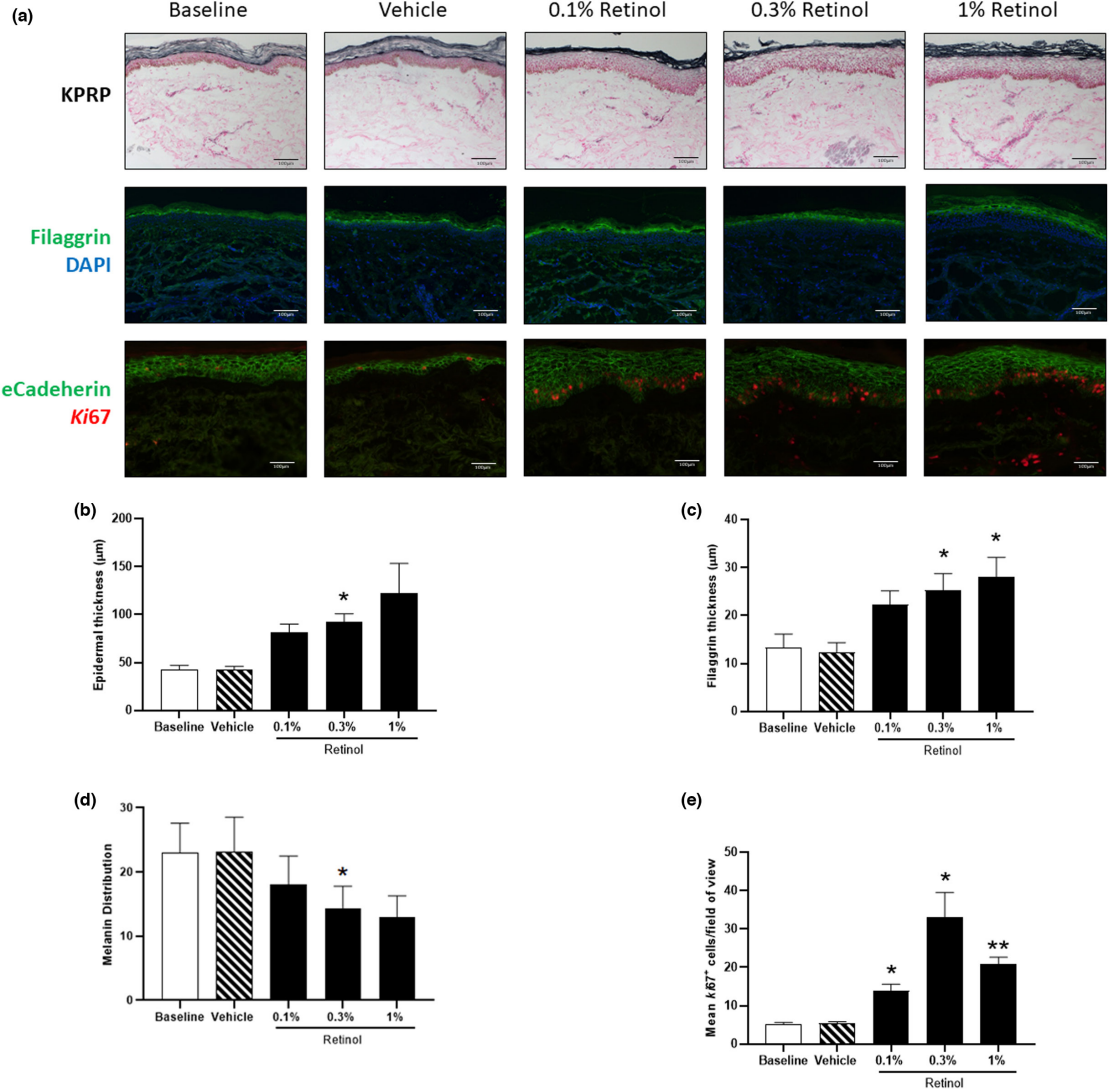
Retinol treatment induces keratinocyte proliferation and expression of proteins required for skin barrier function. (a) Representative images showing immunostaining for keratinocyte proline‐rich protein [KPRP], filaggrin and e‐cadherin/*Ki*67 staining within the epidermis. (b) Quantification of epidermal thickness, (c) filaggrin abundance, (d) melanin distribution and (e) *Ki*67 expression are shown. Statistical significance for differences between treatments compared with the baseline control was assessed by repeated‐measures one‐way ANOVA followed by a Dunnett's multiple comparison test (*p < 0.05, **p < 0.01).

To investigate the effect of retinol on the epidermal barrier structure, immunostaining for filaggrin and KPRP was performed [33, 34]. Due to the fragility of the *stratum corneum* in frozen histological samples, particularly in those treated with retinol, we were unable to perform accurate quantification of these biomarkers across the full depth of the *stratum corneum*. However, we were able to observe a visible increase in the deposition of KPRP within the partially intact *stratum corneum* in response to all concentrations of retinol. Measurement of the depth of filaggrin immunostaining from the *stratum granulosum*, descending towards the *stratum basale*, was also possible (Figure 1a).

Treatment of photoaged skin with retinol induced a dose‐dependent thickening of the epidermis (mean ± SEM; baseline, 42.3 μm ± 4.8; vehicle control, 42.4 μm ± 3.5; 0.1% retinol, 81.62 μm ± 8.5; 0.3% retinol, 92.7 μm ± 8.2; 1% retinol, 122.3 μm ± 31.2; Figure 1b); DEJ convolution remained unaltered across all treatments (data not shown). A concentration‐dependent increase in positive filaggrin staining occurred in response to 0.1% retinol (mean ± SEM; 22.3 μm ± 2.8) and reached levels of statistical significance with 0.3% (25.3 μm ± 3.4, *p* < 0.05) and 1% retinol (28.0 μm ± 4.1, *p* < 0.05) compared to baseline samples (13.2 μm ± 2.9), with the vehicle control having no effect (12.4 μm ± 2.0; Figure 1c). As topical retinoids have been shown to reduce ultraviolet radiation‐induced hyperpigmentation, we further analyzed melanin distribution; here, a concentration‐dependent reduction in melanin coverage was detected in the epidermis following treatment with significance observed at 0.3% retinol (mean ± SEM; baseline, 23.0% ± 4.6; vehicle control, 23.1% ± 5.4; 0.1% retinol, 18.1% ± 4.4; 0.3% retinol, 14.4% ± 3.4; 1% retinol, 13.0% ± 3.6) (Figure 1d).

To determine if other epidermal changes occurred, co‐immunostaining was performed for e‐cadherin (to visualize cell‐to‐cell junctions) and *Ki*67, a protein that accumulates intracellularly during mitosis (Figure 1a). Keratinocyte proliferation as determined by *Ki*67 expression was increased in response to 0.1% retinol (Figure 1e; cells/field of view, mean ± SEM; 14.0 ± 1.6) which became significant following treatment with 0.3% (33.0 ± 6.6, *p* < 0.05) and 1% retinol products (20.8 ± 1.8, *p* < 0.01). The expression of *Ki*67 was unaffected by treatment with vehicle control. The rate of apoptosis was further investigated, but no increase in caspase‐3‐positive cells was observed, regardless of treatment (data not shown). Expression of e‐cadherin was significantly reduced following retinol treatment, regardless of concentration (Figure 1a). Having established that retinol, particularly at the higher doses (0.3% and 1%), influenced the epidermis by inducing keratinocyte proliferation, we investigated the remodelling of dermal ECM components.

**FIGURE 2:**
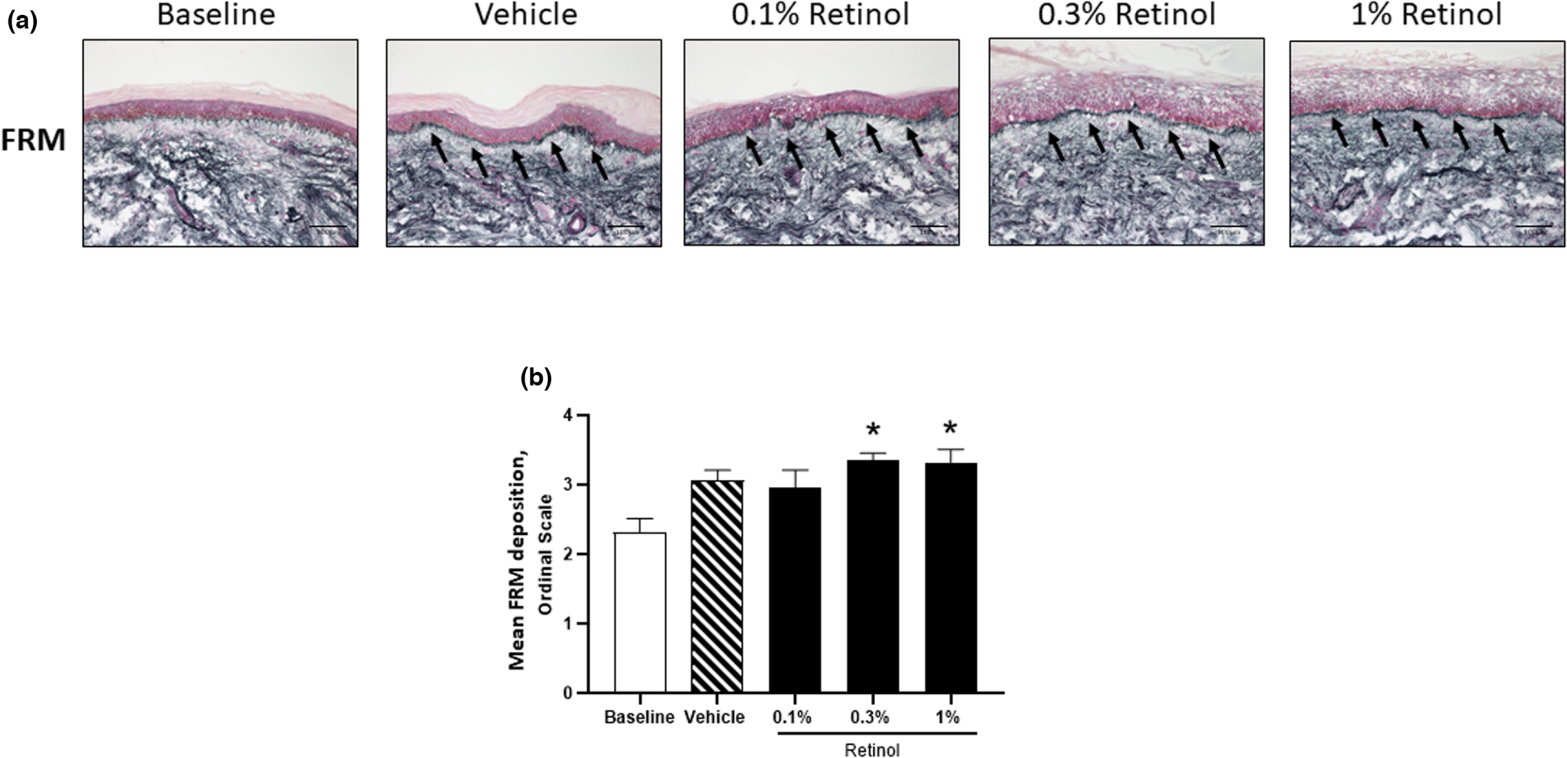
Retinol (0.3%) induces significant deposition of fibrillin‐rich microfibril within the papillary dermis. (a) Representative images showing immunostaining for fibrillin‐rich microfibrils at the papillary dermis. (b) Quantification of fibrillin‐rich microfibril deposition data, presented as the mean ± SEM. Statistical significance for differences between the treatments compared to the baseline control was assessed by repeated‐measures one‐way ANOVA followed by a Dunnett's multiple comparison test (*p < 0.05).

We performed immunostaining for FRM and quantified their abundance within the papillary dermis (Figure 2). Both vehicle and 0.1% retinol occlusion resulted in a small increase in FRM over baseline values (mean ± SEM; baseline, 2.3 ± 0.2; vehicle, 3.1 ± 0.2; 0.1% retinol, 3.0 ± 0.3; Figure 2a), reaching levels of significance in response to both 0.3% (3.6 ± 0.1, *p* < 0.01) and 1% retinol products (3.3 ± 0.2, *p* < 0.05). No changes in global elastin, fibronectin or collagen (mature or newly synthesized) were observed, in agreement with previous short‐term patch test studies [24].

Effectiveness of over‐the‐counter retinol products in ameliorating the clinical signs of photoageing relies upon consumer compliance to long‐term treatment regimens. A retinol tolerance study was therefore designed to safely and as closely as possible reflect the consumer experience of retinol formulation application, tolerance and compliance at home. Female participants were asked to apply either a 0.3% (cohort one; *n* = 115) or 1% (cohort two; *n* = 103) retinol formulation to their face at home for six weeks, gradually increasing the number of applications to once nightly for the final fortnight. These higher concentrations were chosen based on their effectiveness at remodelling the cutaneous microenvironment and their likelihood of causing tolerance issues due to their potency. Participants were asked to self‐report any skin reactions and to grade them as either mild, moderate or severe (Table 2) on a weekly basis.

**FIGURE 3:**
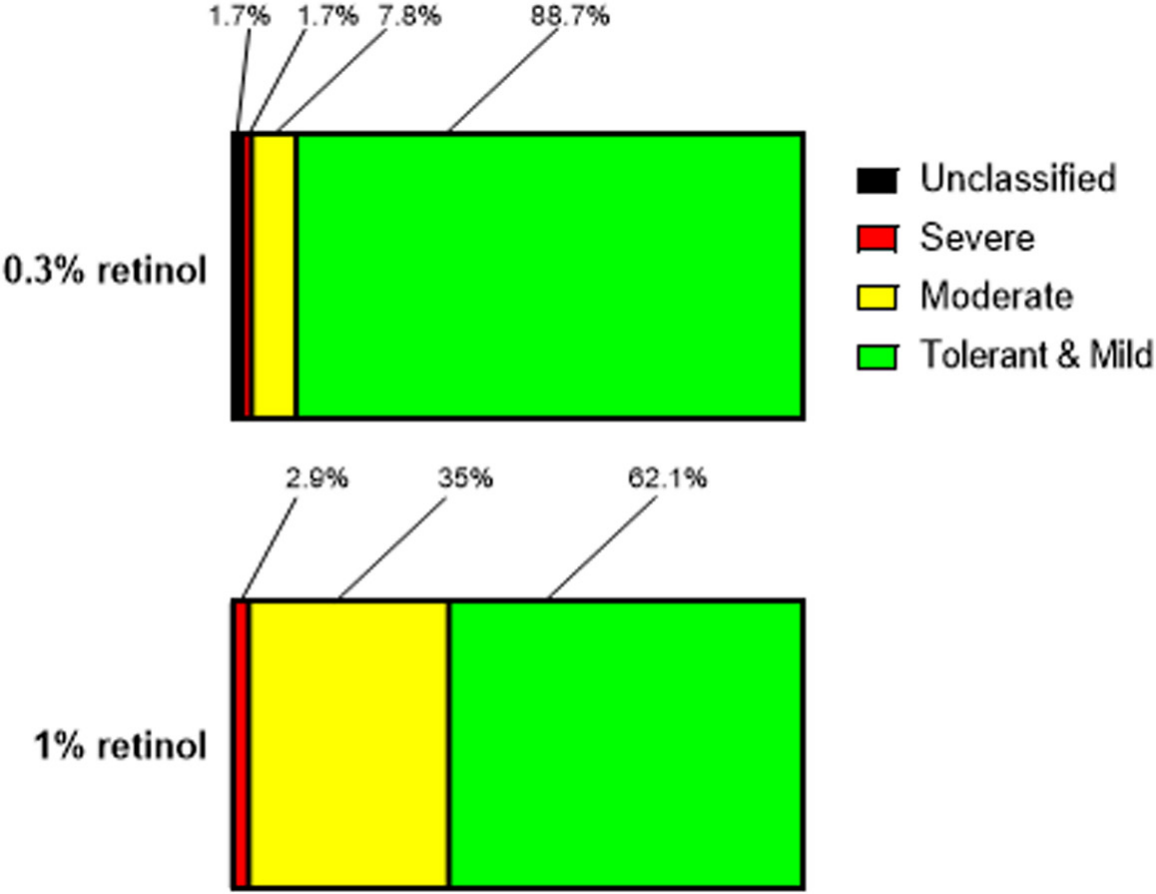
Superior tolerance of 0.3% and 1% retinol by consumers using a daily un‐use regimen. Consumer self‐reported tolerance profiles of individuals applying 0.3% (*n* = 115) and 1% (*n* = 103) retinol formulations to the face in a six‐week regimen. Data shows the percentage of individuals reporting their reactions to the formulations as mild, moderate or severe. Participants reporting no reactions were considered fully tolerant to the retinol formulations. These individuals were categorized with those reporting mild reactions that were considered to fall within the scope of acceptable responses to a topical retinol skin care regimen.

Four participants failed to complete from the 0.3% formulation cohort as compared with 23 individuals from the 1% retinol cohort due to declared tolerance issues. In the 0.3% retinol cohort, 80 of the volunteers (69.6%) reported no reactions, with a further 22 (19.1%) reporting only mild reactions (Figure 3). Such mild reactions were considered expected and tolerable for an over‐the‐counter retinol cosmetic product, based on participant feedback, compliance and expert review by a dermatologist, and so these participants were categorized as ‘tolerant and mild’ (88.7%). In contrast, only 41 volunteers (39.8%) in the 1% cohort reported no skin reactions following application, with a further 23 (22.3%) reporting mild reactions. As before, the latter group of volunteers were also categorized as tolerant and mild (62.1%).

In the 0.3% retinol cohort, nine volunteers (7.8%) reported having a moderate reaction and two (1.7%) reported having a severe reaction, with two of the volunteers (1.7%) in this cohort having reactions that were ‘unclassified’ as no severity information was received from the participant. These two volunteers were excluded from statistical analysis as they could not be assigned to a reaction severity group. Moderate reactions included extensive areas of dryness or skin that was red and sore to the touch. Severe reactions included skin that was red and sore without touching and more persistent reactions including broken skin or blistering, as per the classification guidelines. With the 1% retinol formulation however, 36 volunteers (35%) reported having a moderate reaction and 3 (2.9%) reported having a severe reaction.

Differences between the two cohorts (excluding the *n* = 2 unclassified volunteers) were statistically significant (chi‐square analysis of 0.3% and 1% cohort, tolerant and mild vs moderate and severe; *χ* ^2^(1) = 23.97, *p* < 0.0001) with a trend towards improved tolerant and mild reactions in the 0.3% cohort compared to the 1% retinol cohort.

## DISCUSSION

Using an *in vivo* patch test protocol, we establish that retinol at concentrations >0.1% w/w induces histological remodelling of both the epidermis and dermis of photoaged skin. Further to this, a home‐use, human volunteer escalation study was performed to assess tolerance of the higher concentrations (0.3% and 1%) of retinol. We found that 0.3% retinol was better tolerated than 1%, with less severe reactions when they did occur. Several studies support lowering the concentration of retinol in cosmetic products to reduce problems with skin irritation [26, 35, 36, 37, 38]. One study found clinical benefit in visible wrinkles and evenness of skin tone with 0.3% and 0.5% retinol, the latter concentration being associated with greater irritancy [37]. Similarly, a recent 12‐week dose escalation study reported that a weekly application of a 0.25% retinol formulation, gradually increasing to three times per week, also improved the clinical appearance of photoaged facial skin [26]. Analysis of our MPT biopsies found that 0.3% was comparable to 1% retinol in terms of remodelling dermal ECM components and inducing rapid epidermal changes. Importantly, this bioactivity of 0.3% retinol was combined with a significant improvement in consumer tolerance compared to 1%.

The *in vivo* patch test protocol used here allows for assessment of cutaneous change in a relatively short period of time (up to 12 days), as opposed to in‐use studies where products may only provide evidence of remodelling at time points >3‐months; hence, it is a useful tool for assessing potential benefit within a product's development pipeline, ahead of any longer‐term clinical studies [25]. In this study, epidermal architecture in photodamaged skin was remodelled by retinol, regardless of its concentration, with increased expression of filaggrin and KPRP. The depth of filaggrin, distributed through the epidermis, increased in a concentration‐dependent manner, concomitant with epidermal expansion. Although the functional integrity of the barrier can be affected by such dynamic changes [39, 40, 41], published literature supports its stabilization over time with prolonged product use [42]. We further observed a concentration‐dependent reduction in melanin, which may be related to keratinocyte proliferation. Rapid epidermal expansion may result in a greater distribution of melanin throughout epidermal layers and explain why a retinol‐induced reduction of seasonal melanin distribution to suprabasal keratinocyte layers has been reported in the literature [43, 44]. However, others have shown that retinol causes depigmentation of skin via the downregulation of proteins such as tyrosinase and tyrosinase‐related protein, known to regulate melanogenesis [45, 46, 47, 48], although evidence of the direct suppressing effects of ATRA on melanogenesis has not been demonstrated *in vitro* [43]. Downregulation of e‐cadherin in response to retinol may further expedite keratinocyte differentiation and transition through the *strata* of the epidermis towards the *stratum corneum*. Compared to the other concentrations, 0.3% retinol‐treated skin had the highest mean number of *Ki*67^+^ cells and the lowest level of e‐cadherin expression. Proliferation of basal keratinocytes unaffected the rate of apoptosis, suggesting that 1% retinol induced rapid epidermal expansion before reaching homeostasis. This dynamic epidermal expansion may occur more gradually, or may be sustained for longer, with 0.1% and 0.3% rather than 1% retinol.

Consistent with previously published data from our laboratory [25, 30, 49], *de novo* FRM synthesis was induced with 0.3% and 1% retinol. The levels of FRM deposition were greater with 0.3% and 1% retinol than with vehicle alone. Fibrillin‐rich microfibrils are essential for elastogenesis and development of mature elastic fibres [50], whilst fibronectin is required for FRM assembly [51]. However, despite increased FRM deposition, global cutaneous elastin and fibronectin networks were unaltered. Likewise, mature collagens I, III and VII remained unchanged, possibly due to short product application time, in comparison to longer occlusion studies [25, 52].

Tolerance of a retinol‐containing product cannot be wholly assessed using an *in vivo* patch test, although erythema and *stratum corneum* integrity and/or dryness can be visualized in some volunteers on patch removal. It is therefore appropriate to perform tolerance‐profile studies on cohorts of potential consumers for assessment of any unwanted side effects in the longer term. Consistent with others, our escalating use tolerance‐profile study suggests that the concentration of topical retinol used impacts an individual's ability to use a retinol‐containing over‐the‐counter product mainly due to lack of tolerance [26, 37]. Greater tolerance towards 0.3% retinol, compared with the 1% formulation, was associated with fewer and milder skin reactions. These mild reactions were managed appropriately by the participants and were not considered to be of greater severity than those expected for a retinol product currently available on the skincare market. However, this regimen, like others [26], used a gradual escalation of application. The application of the retinol was combined with the diligent use of a night cream and SPF day cream, both also being important in improving tolerance. Topical over‐the‐counter retinol products therefore require clear guidance on their proper use to minimize skin sensitization.

Although the dynamics of the skin's response to concentrations of retinol was not investigated in the current in‐use study, the epidermal remodelling occurring suggests that 1% retinol drives a rapid retinoid response, whilst a more gradual response, associated with fewer skin irritancy issues, occurred with 0.3% retinol. Therefore, whilst a 1% product may satisfy consumer demand for rapid amelioration of the photoaged phenotype, long‐term effective treatment of photoaged skin with 0.3% retinol may be the preferred product choice for individuals where retinol sensitivity has previously been an issue. This supports previously published data, where 0.025% ATRA was shown to provide similar benefit to photoaged skin to 0.1% ATRA, but with markedly less irritation over the 48‐week study period [53], and supports the hypothesis that irritancy is not solely required for retinoid efficacy. A time‐dependent long‐term study to assess the clinical benefits of this cosmetic formulation of retinol to photoaged skin is therefore warranted to assess the potential of this over‐the‐counter product's capacity for providing an anti‐ageing skin benefit.

## CONFLICT OF INTEREST

None.

## References

[1] Langton AK , Ayer J , Griffiths TW , Rashdan E , Naidoo K , Caley MP , et al. Distinctive clinical and histological characteristics of atrophic and hypertrophic facial photoageing. J Eur Acad Dermatol Venereol. 2021;35(3):762–8. 33275818 10.1111/jdv.17063 PMC7986784

[2] Kligman AM . Early destructive effect of sunlight on human skin. JAMA. 1969;210(13):2377–80. 5395389

[3] Cerimele D , Celleno L , Serri F . Physiological changes in ageing skin. Br J Dermatol. 1990;122(Suppl 35):13–20. 10.1111/j.1365-2133.1990.tb16120.x 2186780

[4] Newton VL , Bradley RS , Seroul P , Cherel M , Griffiths CEM , Rawlings AV , et al. Novel approaches to characterize age‐related remodelling of the dermal‐epidermal junction in 2D, 3D and *in vivo* . Skin Res Technol. 2017;23(2):131–48. 27502896 10.1111/srt.12312

[5] Tsuji T . Scanning electron microscope studies of solar elastosis. Br J Dermatol. 1980;103(3):307–12. 7426427 10.1111/j.1365-2133.1980.tb07249.x

[6] Watson REB , Griffiths CEM , Craven NM , Shuttleworth CA , Kielty CM . Fibrillin‐rich microfibrils are reduced in photoaged skin. Distribution at the dermal‐epidermal junction. J Invest Dermatol. 1999;112(5):782–7. 10233772 10.1046/j.1523-1747.1999.00562.x

[7] Dalle Carbonare M , Pathak MA . Skin photosensitizing agents and the role of reactive oxygen species in photoaging. J Photochem Photobiol B. 1992;14(1–2):105–24. 1331386 10.1016/1011-1344(92)85086-a

[8] Avery NC , Bailey AJ . The effects of the Maillard reaction on the physical properties and cell interactions of collagen. Pathol Biol (Paris). 2006;54(7):387–95. 16962252 10.1016/j.patbio.2006.07.005

[9] Langton AK , Graham HK , Griffiths CEM , Watson REB . Ageing significantly impacts the biomechanical function and structural composition of skin. Exp Dermatol. 2019;28(8):981–4. 31152614 10.1111/exd.13980 PMC6851988

[10] Vandiver AR , Hogan SR . Aging skin and non‐surgical procedures: a basic science overview. Plast Aesthet Res. 2020;7:63.

[11] Ascenso A , Ribeiro H , Marques H , Oliveira H , Santos C , Simões S . Is tretinoin still a key agent for photoaging management? Mini Rev Med Chem. 2014;14(8):629–41. 25141855 10.2174/1389557514666140820102735

[12] Kawaguchi R , Yu J , Honda J , Hu J , Whitelegge J , Ping P , et al. A membrane receptor for retinol binding protein mediates cellular uptake of vitamin A. Science. 2007;315(5813):820–5. 17255476 10.1126/science.1136244

[13] Sun H , Kawaguchi R . The membrane receptor for plasma retinol‐binding protein, a new type of cell‐surface receptor. Int Rev Cell Mol Biol. 2011;288:1–41. 21482409 10.1016/B978-0-12-386041-5.00001-7 PMC3907177

[14] Aström A , Tavakkol A , Pettersson U , Cromie M , Elder JT , Voorhees JJ . Molecular cloning of two human cellular retinoic acid‐binding proteins (CRABP). Retinoic acid‐induced expression of CRABP‐II but not CRABP‐I in adult human skin *in vivo* and in skin fibroblasts in vitro. J Biol Chem. 1991;266(26):17662–6. 1654334

[15] Eller MS , Oleksiak MF , McQuaid TJ , McAfee SG , Gilchrest BA . The molecular cloning and expression of two CRABP cDNAs from human skin. Exp Cell Res. 1992;198(2):328–36. 1309505 10.1016/0014-4827(92)90387-n

[16] Petkovich M , Brand NJ , Krust A , Chambon P . A human retinoic acid receptor which belongs to the family of nuclear receptors. Nature. 1987;330(6147):444–50. 2825025 10.1038/330444a0

[17] Brand N , Petkovich M , Krust A , Chambon P , de Thé H , Marchio A , et al. Identification of a second human retinoic acid receptor. Nature. 1988;332(6167):850–3. 2833708 10.1038/332850a0

[18] Fisher GJ , Talwar HS , Xiao JH , Datta SC , Reddy AP , Gaub MP , et al. Immunological identification and functional quantitation of retinoic acid and retinoid X receptor proteins in human skin. J Biol Chem. 1994;269(32):20629–35. 8051161

[19] Xiao JH , Durand B , Chambon P , Voorhees JJ . Endogenous retinoic acid receptor (RAR)‐retinoid X receptor (RXR) heterodimers are the major functional forms regulating retinoid‐responsive elements in adult human keratinocytes. Binding of ligands to RAR only is sufficient for RAR‐RXR heterodimers to confer ligand‐dependent activation of hRAR beta 2/RARE (DR5). J Biol Chem. 1995;270(7):3001–11. 7852380 10.1074/jbc.270.7.3001

[20] Kurlandsky SB , Xiao JH , Duell EA , Voorhees JJ , Fisher GJ . Biological activity of all‐trans retinol requires metabolic conversion to all‐trans retinoic acid and is mediated through activation of nuclear retinoid receptors in human keratinocytes. J Biol Chem. 1994;269(52):32821–7. 7806506

[21] Watson REB , Gibbs NK , Griffiths CE , Sherratt MJ . Damage to skin extracellular matrix induced by UV exposure. Antioxid Redox Signal. 2014;21(7):1063–77. 24124905 10.1089/ars.2013.5653

[22] Naylor EC , Watson REB , Sherratt MJ . Molecular aspects of skin ageing. Maturitas. 2011;69(3):249–56. 21612880 10.1016/j.maturitas.2011.04.011

[23] Lee CM . Fifty years of research and development of cosmeceuticals: a contemporary review. J Cosmet Dermatol. 2016;15(4):527–39. 27496663 10.1111/jocd.12261

[24] Kong R , Cui Y , Fisher GJ , Wang X , Chen Y , Schneider LM , et al. A comparative study of the effects of retinol and retinoic acid on histological, molecular, and clinical properties of human skin. J Cosmet Dermatol. 2016;15(1):49–57. 26578346 10.1111/jocd.12193

[25] Watson REB , Craven NM , Griffiths CEM , Kang S , Jones CJP , Kielty CM . A short‐term screening protocol, using fibrillin‐1 as a reporter molecule, for photoaging repair agents. J Invest Dermatol. 2001;116(5):672–8. 11348454 10.1046/j.1523-1747.2001.01322.x

[26] Draelos ZD , Peterson RS . A double‐blind, comparative clinical study of newly formulated retinol serums vs tretinoin cream in escalating doses: a method for rapid retinization with minimized irritation. J Drugs Dermatol. 2020;19(6):625–31. 32574009 10.36849/JDD.2020.10.36849/JDD.2020.5085

[27] Kim BH , Lee YS , Kang KS . The mechanism of retinol‐induced irritation and its application to anti‐irritant development. Toxicol Lett. 2003;146(1):65–73. 14615068 10.1016/j.toxlet.2003.09.001

[28] Temova Rakusa Z , Škufca P , Kristl A , Roškar R . Quality control of retinoids in commercial cosmetic products. J Cosmet Dermatol. 2020;20:1166–75. 32813932 10.1111/jocd.13686

[29] Joly‐Tonetti N , Wibawa JID , Bell M , Tobin D . Melanin fate in the human epidermis: a reassessment of how best to detect and analyse histologically. Exp Dermatol. 2016;25(7):501–4. 26998907 10.1111/exd.13016

[30] Watson REB , Long SP , Bowden JJ , Bastrilles JY , Barton SP , Griffiths CE . Repair of photoaged dermal matrix by topical application of a cosmetic 'antiageing' product. Br J Dermatol. 2008;158(3):472–7. 18070204 10.1111/j.1365-2133.2007.08364.x

[31] Schindelin J , Arganda‐Carreras I , Frise E , Kaynig V , Longair M , Pietzsch T , et al. Fiji: an open‐source platform for biological‐image analysis. Nat Methods. 2012;9(7):676–82. 22743772 10.1038/nmeth.2019 PMC3855844

[32] Timár F , Soós G , Szende B , Horváth A . Interdigitation index—a parameter for differentiating between young and older skin specimens. Skin Res Technol. 2000;6(1):17–20. 11428937 10.1034/j.1600-0846.2000.006001017.x

[33] Sandilands A , Sutherland C , Irvine AD , McLean WHI . Filaggrin in the frontline: role in skin barrier function and disease. J Cell Sci. 2009;122(Pt 9):1285–94. 19386895 10.1242/jcs.033969 PMC2721001

[34] Suga H , Oka T , Sugaya M , Sato Y , Ishii T , Nishida H , et al. Keratinocyte proline‐rich protein deficiency in atopic dermatitis leads to barrier disruption. J Invest Dermatol. 2019;139(9):1867–75.e7. 30905808 10.1016/j.jid.2019.02.030

[35] Gold MH , Kircik LH , Bucay VW , Kiripolsky MG , Biron JA . Treatment of facial photodamage using a novel retinol formulation. J Drugs Dermatol. 2013;12(5):533–40. 23652947

[36] Babcock M , Mehta RC , Makino ET . A randomized, double‐blind, split‐face study comparing the efficacy and tolerability of three retinol‐based products vs. three tretinoin‐based products in subjects with moderate to severe facial photodamage. J Drugs Dermatol. 2015;14(1):24–30. 25607905

[37] Zasada M , Budzisz E , Erkiert‐Polguj A . A clinical anti‐ageing comparative study of 0.3 and 0.5% retinol serums: a clinically controlled trial. Skin Pharmacol Physiol. 2020;33(2):102–16. 32428912 10.1159/000508168

[38] Tucker‐Samaras S , Zedayko T , Cole C , Miller D , Wallo W , Leyden JJ . A stabilized 0.1% retinol facial moisturizer improves the appearance of photodamaged skin in an eight‐week, double‐blind, vehicle‐controlled study. J Drugs Dermatol. 2009;8(10):932–6. 19852122

[39] Elias PM , Fritsch PO , Lampe M , Williams ML , Brown BE , Nemanic M , et al. Retinoid effects on epidermal structure, differentiation, and permeability. Lab Invest. 1981;44(6):531–40. 6939940

[40] Chen S , Ostrowski J , Whiting G , Roalsvig T , Hammer L , Currier SJ , et al. Retinoic acid receptor gamma mediates topical retinoid efficacy and irritation in animal models. J Invest Dermatol. 1995;104(5):779–83. 7738355 10.1111/1523-1747.ep12606988

[41] Thacher SM , Standeven AM , Athanikar J , Kopper S , Castilleja O , Escobar M , et al. Receptor specificity of retinoid‐induced epidermal hyperplasia: effect of RXR‐selective agonists and correlation with topical irritation. J Pharmacol Exp Ther. 1997;282(2):528–34. 9262312

[42] Ellis CN , Weiss JS , Hamilton TA , Headington JT , Zelickson AS , Voorhees JJ . Sustained improvement with prolonged topical tretinoin (retinoic acid) for photoaged skin. J Am Acad Dermatol. 1990;23(Pt 1):629–37. 2229490 10.1016/0190-9622(90)70265-j

[43] Yoshimura K , Tsukamoto K , Okazaki M , Virador VM , Lei TC , Suzuki Y , et al. Effects of all‐trans retinoic acid on melanogenesis in pigmented skin equivalents and monolayer culture of melanocytes. J Dermatol Sci. 2001;27(Suppl 1):S68–75. 11514127 10.1016/s0923-1811(01)00116-5

[44] Tancrède‐Bohin E , Baldeweck T , Brizion S , Decencière E , Victorin S , Ngo B , et al. *In vivo* multiphoton imaging for non‐invasive time course assessment of retinoids effects on human skin. Skin Res Technol. 2020;26(6):794–803. 32713074 10.1111/srt.12877 PMC7754381

[45] Talwar H , Griffiths CE , Fisher GJ , Russman A , Krach K , Benrazavi S , et al. Differential regulation of tyrosinase activity in skin of white and black individuals *in vivo* by topical retinoic acid. J Invest Dermatol. 1993;100(6):800–5. 8496619 10.1111/1523-1747.ep12476615

[46] Sorg O , Kasraee B , Salomon D , Saurat JH . The potential depigmenting activity of retinaldehyde. Dermatology. 2013;227(3):231–7. 24080511 10.1159/000354294

[47] Romero C , Aberdam E , Larnier C , Ortonne JP . Retinoic acid as modulator of UVB‐induced melanocyte differentiation. Involvement of the melanogenic enzymes expression. J Cell Sci. 1994;107(Pt 4):1095–103. 8056833 10.1242/jcs.107.4.1095

[48] Kasraee B , Fallahi MR , Ardekani GS , Ebrahimi S , Doroudchi G , Omrani GR , et al. Retinoic acid synergistically enhances the melanocytotoxic and depigmenting effects of monobenzylether of hydroquinone in black Guinea pig skin. Exp Dermatol. 2006;15(7):509–14. 16761959 10.1111/j.1600-0625.2006.00441.x

[49] Watson REB , Ogden S , Cotterell LF , Bowden JJ , Bastrilles JY , Long SP , et al. Effects of a cosmetic 'anti‐ageing' product improves photoaged skin. Br J Dermatol. 2009;161(2):419–26. 19438432 10.1111/j.1365-2133.2009.09216.x PMC2774146

[50] Trask TM , Trask BC , Ritty TM , Abrams WR , Rosenbloom J , Mecham RP . Interaction of tropoelastin with the amino‐terminal domains of fibrillin‐1 and fibrillin‐2 suggests a role for the fibrillins in elastic fiber assembly. J Biol Chem. 2000;275(32):24400–6. 10825173 10.1074/jbc.M003665200

[51] Kinsey R , Williamson MR , Chaudhry S , Mellody KT , McGovern A , Takahashi S , et al. Fibrillin‐1 microfibril deposition is dependent on fibronectin assembly. J Cell Sci. 2008;121(Pt 16):2696–704. 18653538 10.1242/jcs.029819

[52] Craven NM , Watson RE , Jones CJ , Shuttleworth CA , Kielty CM , Griffiths CE . Clinical features of photodamaged human skin are associated with a reduction in collagen VII. Br J Dermatol. 1997;137(3):344–50. 9349327

[53] Griffiths CEM , Kang S , Ellis CN , Kim KJ , Finkel LJ , Ortiz‐Ferrer LC , et al. Two concentrations of topical tretinoin (retinoic acid) cause similar improvement of photoaging but different degrees of irritation. A double‐blind, vehicle‐controlled comparison of 0.1% and 0.025% tretinoin creams. Arch Dermatol. 1995;131(9):1037–44. 7544967

